# Job satisfaction of graduates of rural oriented medical students training project in Jiangsu Province, China: a cross-sectional study

**DOI:** 10.1186/s12909-021-03074-z

**Published:** 2022-01-03

**Authors:** Wenjun Yan, Xiuyin Gao, Wei Wang, Zhengyu Zhou, Chao Zou, Zhaojun Lu

**Affiliations:** 1 Department of General Practice, School of Public Health, Xuzhou Medical University, Xuzhou, 221000 Jiangsu China; 2 West-City Central Hospital, Lianyungang, 222100 Jiangsu China; 3 Ma-an Community Health Centre, Nanjing, 211500 Jiangsu China; 4 Second Clinical Medical College, Xuzhou Medical University, Xuzhou, 221000 Jiangsu China

**Keywords:** Graduates of rural oriented medical students training project, rural physicians, Job satisfaction, Survey

## Abstract

**Background:**

The Chinese government has worked out the “Rural Oriented Medical Students Training Project” to address physician maldistribution, which attempted to train physicians for rural areas. The present study attempted to evaluate the job satisfaction of the graduates of this project in Jiangsu Province, China.

**Methods:**

Online questionnaires were sent to the graduates of the “Rural Oriented Medical Students Training Project” (group A) and their colleagues, who were rural physicians recruited from different sources (group B). The study was approved by the Ethics Committee of Xuzhou Medical University, and the approval number was 2,018,057. Information on demographic characteristics, work conditions, and self-reported satisfaction was collected to compare the satisfaction differences between the two recruited rural physicians using the Chi-square test and Mann–Whitney U test. Additionally, factors correlated to the satisfaction of group A were assessed using multivariate linear regression. Statistical analysis was performed using SPSS 23.0 (SPSS Inc., Chicago, IL, USA). *P* < 0.05 was considered statistically significant.

**Results:**

Group A exhibited moderate satisfaction (2.81 ± 0.687). The satisfaction score from the highest to the lowest was for occupational ecology, life satisfaction, stress, competency, and internal environment. Positive factors related to the satisfaction of group A were area, monthly income, working hours per week, professional title, and post.

**Conclusion:**

The satisfaction of the graduates of the “Rural Oriented Medical Students Training Project” was moderate. Factors related to satisfaction included economic incentives, workload, and professional confidence. Possible solutions for increasing satisfaction should consist of economic support and possible ways to improve the professional identification of these graduates.

## Introduction

Physicians tend to settle in urban areas, creating a worldwide shortage of rural physicians [1]. This further limits the improvement of medical quality in rural areas. In China, traditional medical education produces few rural physicians. One solution to overcome this problem is to train more physicians who identify with and desire to practice among rural communities [2]. Several studies referred to rural upbringing and the choice of family medicine as a specialization as factors strongly associated with rural practice [3], suggesting that students with a rural background, rural practice intent, or more excellent service orientation should be recruited into rural practice [4]. Greater rural exposure through either upbringing or education motivates future rural practice and generates a sense of familiarity and community involvement [5].

Numerous countries have initiated solutions and projects to recruit more rural doctors and improve their retention rate. These include “Physician Shortage Area Program (PSAP) [6]” and “Rural Medical Scholars Program (RMSP) [7]” in America, “Rural Clinical School (RCS) Program [8]” in Australia, “Jichi Medical University (JMU) Program [9]” in Japan, “Collaborative Project to Increase Production of Rural Doctors (CPIRD) [10]” in Thailand, and “the Friends of Mosveld Scholarship Scheme (FOMSS) [11]” in South Africa. In China, the plan of “Healthy China 2030” proposed a focus on general practitioners, making it essential to increase the staff in rural and remote areas [12]. The Chinese government introduced the “Rural Oriented Medical Students Training Project [(2010) No. 1198]” in 2010 to deal with the severe shortage of rural physicians. This project attempted to train at least 5000 rural physicians for free annually. According to this policy, the medical colleges in each province recruit and train local rural students annually. Before entering school, the rural students are required to sign contracts with their local bureaus of sanitation. According to the agreement, the graduates must return to their hometown after receiving free medical education and serve as rural physicians for at least 6 years.

Because Jiangsu ranks among the top provinces in terms of gross domestic product (GDP) and per capita GDP in China, it played a leading role in several aspects, including the medical industry. Jiangsu was also one of the earliest provinces to follow the “Rural Oriented Medical Students Training Project”. About two hundred of graduates of this project are initiated into the rural medical ecosystem every year.

In order to solve the shortage of doctors in rural areas, on the one hand, rural doctors should be constantly recruited, on the other hand, the retention rate of existing doctors should be ensured. Most of the studies in China and abroad focused on increasing the number of rural doctors. however, rural doctors’ retention willing is not clear, questions such as “Are they satisfied with their job as rural physicians?” and “Are they optimistic about being rural physicians?” must be answered and are highly relevant to policymakers. Thus, the present study attempted to evaluate the satisfaction of the graduates of this project in the Jiangsu Province and identify factors associated with higher levels of career satisfaction.

## Methods

### Design

The present cross-sectional survey was conducted on 507 physicians working in the rural sector from April 2018 to August 24, 2019, under the guidelines of the STROBE statement. A multistage stratified sampling method was employed to select the respondents in the study. Sampling rules were established in the first stage. Jiangsu is an imbalanced province due to the economic level of the 13 cities. The province can be divided into three parts according to financial status, namely northern Jiangsu (per capita GDP = 75,551 RMB), which includes Xuzhou, Lianyungang, Yancheng, Huaian, and Suqian; central Jiangsu (per capita GDP = 123,551 RMB), which includes Yangzhou, Nantong, and Taizhou; and southern Jiangsu (per capita GDP = 167,995 RMB), which includes Suzhou, Wuxi, Changzhou, Zhenjiang, and Nanjing [13]. Because the retention rate of doctors is related to economic level [14], two cities were selected randomly in northern, central, and southern Jiangsu by drawing lots. Lianyungang and Yancheng were chosen from northern Jiangsu, Yangzhou and Nantong were selected from central Jiangsu, and Nanjing and Changzhou were selected from southern Jiangsu. Our university recruited rural medical students from all 13 cities in Jiangsu, and there was a name list of graduates for each city.

In the second stage, top two graduates for each chosen city were selected from the name list to be liaisons. If the two liaisons in one city worked in the same hospital, skipped the second one until met the one who worked in a different hospital, a total of 12 liaisons were selected. All the liaisons were asked to attend online training and send questionnaires to their rural physician colleagues through their WeChat working group. Team leaders were asked to inform all the respondents of the purpose of this survey before sending questionnaires. To increase the response rate, all respondents received 5 RMB when they handed in their questionnaires. Thus, a series of information was collected. The participants were divided into two groups: group A comprising graduates of the “Rural Oriented Medical Students Training Project” and group B (control group) comprising rural physicians who were not recruited from the project. The survey was conducted on August 15, 2018 for the first time and on August 21, 2019 for the second time. To ensure maximum participation of rural doctors, team leaders connected with doctors who did not respond in WeChat groups in 1 week and asked them to complete the questionnaires. The survey ended on August 24, 2019.

### Questionnaire

The questionnaire was evaluated and approved by a team of rural health specialists that included experienced doctors and epidemiologists before being sent to the rural physicians. Data on the demographic characteristics and work conditions were collected, whereas self-scale satisfaction was evaluated. All self-evaluation items of satisfaction were classified into five categories by principal component analysis (PCA) (Box 1) comprising internal environment, comfort, competency, stress, and occupational ecology. Questions about satisfaction were self-evaluated using the following five-point scale: 1: dissatisfied; 2: relatively dissatisfied; 3: moderate; 4: relatively satisfied; 5: satisfied. The Cronbach’s α coefficient was 0.929, whereas the Kaiser–Meyer–Olkin (KMO) was 0.926. The significance of Bartlett’s test of sphericity was *P* < 0.05. The questionnaire exhibited superior reliability and validity..

**Box 1:** Satisfaction Scales

	Composition				
	Internal Environment	Comfort	Competency	Stress	Occupational ecology
How satisfied with the welfare in your hospital?	0.767				
How satisfied with your employment?	0.756				
How satisfied with your social insurance?	0.746				
How satisfied with your income?	0.669				
How satisfied with your own promotion space?	0.640				
How satisfied with your own training opportunities?	0.605				
How satisfied with the relationship with your colleagues?	0.604				
How satisfied with your work environment?	0.603				
How satisfied with the balance of income and contribution?	0.591				
How satisfied with the implementation of policies?	0.558				
How satisfied with your living convenience?		0.694			
How satisfied with your job?		0.682			
How satisfied with your life?		0.674			
How satisfied with your living conditions?		0.591			
How satisfied with your career?		0.540			
How satisfied with your professional skills?			0.853		
How satisfied with your professional knowledge?			0.848		
How satisfied with your learning ability?			0.795		
How satisfied with your motivation?			0.588		
How satisfied with your Researching capacity?			0.561		
How satisfied with your clinical workload?				−0.814	
How satisfied with your level of stress at work?				0.753	
How satisfied with your public health workload?				−0.581	
How satisfied with the doctor-patient relationships					0.709
How satisfied with your career reputation?					0.630

The following key question was asked to all participants to evaluate the possibility of quitting their position as a rural physician in the future: “Do you intend to work for your current workplace until you retire?”

### Statistical analysis

All data were exported in Excel form from the questionnaire network (www.wjx.cn). The internal consistency of “satisfaction” was assessed by the Cronbach’s α coefficient for the questionnaire. Additionally, the KMO and significance of Bartlett’s test of sphericity were reported. The Kolmogorov–Smirnov test was used to test the normal distribution assumption. The Chi-square test was used to test the differences between groups A and B for binary variables, whereas the Mann–Whitney U test was used for rank variables. The six dimensions of satisfaction were presented as mean and standard deviation (SD). The Mann–Whitney U test was used for comparing satisfaction between the two groups. A multivariate linear regression model was used to estimate fixed effects of covariates on each dimension of satisfaction of group A. Results were presented as unstandardized coefficients B and standardized coefficients Beta in addition to significance. Statistical analyses were performed using SPSS 23.0 (SPSS Inc., Chicago, IL, USA). A *P*-value < 0.05 was considered statistically significant.

## Results

Of the 827 responses received, questionnaires with missing information and misinformation were excluded. Thus, 722 responses were collected (response rate was 87.3%). Because the first graduates of the “Rural Oriented Medical Students Training Project” had just started working in 2012, there would not be any respondents beyond 40 years. Thus, all respondents above 40 years of age were deleted. The effective respondents were 507 (effective response rate was 61.3%)..

### Demographic characteristics

Of the 507 participating physicians, 262 (51.7%) physicians were in group A, whereas 245(48.3%) physicians were in group B. Of the 245 physicians in group A, 45.8% (*n* = 120) came from the northern part of Jiangsu, 80.9% (*n* = 212) were under 30 years old, 55.3% (*n* = 145) were female, 69.8% (*n* = 183) were in a relationship, 59.9% (*n* = 157) earned a monthly income between 3000 and 6000 RMB, 79.4% (*n* = 208) were residents, and 85.9% (*n* = 225) worked as clinicians. As the first class of the “Rural Oriented Medical Students Training Project” just started working in 2012, they were younger and less likely to have a professional title (Table 1).

**Table 1:** Demographic Characteristics of Group A and Group B

		Group A (%)	Group B (%)	χ^2^ or *Z*	*P*
Area	Northern JS	120(45.8)	101(41.2)	−1.650	0.099
	Central JS	77(29.4)	63(25.7)		
	Southern JS	65(24.8)	81(33.1)		
Age	< 30	212(80.9)	106(43.3)	76.76	< 0.001
	30–39	50(19.1)	139(56.7)		
Sex	Male	117(44.7)	96(39.2)	1.557	0.212
	Female	145(55.3)	149(60.8)		
Marriage	Single	79(30.2)	62(25.3)	1.481	0.224
	In a relationship	183(69.8)	183(74.7)		
Income Per month (RMB)	< 3000	63(24.0)	58(23.7)	−0.791	0.429
	3000–6000	157(59.9)	160(65.3)		
	> 6000	42(16.1)	27(11.0)		
Professional title	Resident	208(79.4)	165(67.3)	−3.233	< 0.001
	Attending	54(20.6)	72(29.4)		
	Associate chief physician	0	4(1.6)		
	Chief physician	0	4(1.6)		
Post	Clinician	225(85.9)	204(83.3)	−1.066	0.286
	Public health physician	22(8.4)	12(4.9)		
	TCM physician*	12(4.6)	19(7.8)		
	Dentist	2(0.7)	4(1.6)		
	Administrator	1(0.4)	6(2.4)		

*TCM = Traditional Chinese Medicine

### Work conditions

The top three work duties of the project graduates were outpatient service (80.5%), communication with patients (46.2%), and file work (39.7%). Although 55.3% of graduates attended no more than 60 patients per week, 36.3% of graduates worked for more than 50 h per week. Of the total group A participants, 56.5% wanted further education opportunities. Participants from group A exhibited higher participation in outpatient service (*P* = 0.016) and home visits (*P* = 0.007), higher standardized training rate (*P* < 0.001) (standardized training is regarded as a guarantee of high-quality care in China), higher advanced rank rate (*P* < 0.001), and higher salary in the same rank (*P* < 0.001) than group B (Table 2). In some areas, the recruitment advertisements publicized the possibility of obtaining an advanced rank for graduates of the “Rural Oriented Medical Students Training Project” and higher salaries in the same rank. The data in this investigation concurred with this policy. However, 34.0% of group A were considering quitting their job..

**Table 2:** Work condition of Group A and Group B

			Group A (%)	Group B (%)	χ^2^ or *Z*	*P*
Major work	Outpatient service	No	51(19.5)	70(28.6)	5.777	0.016
		Yes	211(80.5)	175(71.4)		
	File work*	No	158(60.3)	166(67.8)	3.046	0.081
		Yes	104(39.7)	79(32.2)		
	Communication	No	141(53.8)	142(58.0)	0.881	0.348
		Yes	121(46.2)	103(42.0)		
	Home visit	No	169(64.5)	185(75.5)	7.279	0.007
		Yes	93(35.5)	60(24.5)		
	Health education	No	228(87.0)	210(85.7)	0.184	0.668
		Yes	34(13.0)	35(14.3)		
Patients per week	< 30		87(33.2)	87(35.5)	−1.084	0.278
	30~		58(22.1)	63(25.7)		
	60~		42(16.0)	35(14.3)		
	90~		26(9.9)	21(8.6)		
	120~		49(18.7)	39(16.0)		
Work hours per week	< 40		59(22.5)	50(20.4)	−0.820	0.412
	40~		108(41.2)	98(40.0)		
	50~		95(36.3)	97(39.6)		
Standardized training		Yes	207(79.0)	98(40.0)	80.375	< 0.001
		No	55(21.0)	147(60.0)		
Advanced rank		Yes	61(23.3)	21(8.6)	20.217	< 0.001
		No	201(76.7)	224(91.4)		
Higher salary in the same rank		Yes	28(10.7)	4(1.6)	17.550	< 0.001
		No	234(89.3)	241(98.4)		
Plan in the coming 3 years	Unchanged		66(25.2)	41(16.7)	−1.548	0.122
	Further education		148(56.5)	159(64.9)		
	Not a concern		48(18.3)	45(18.4)		
Willing to quit in the future	Yes		89(34.0)	69(28.2)	−0.762	0.446
	No		55(21.0)	64(26.1)		
	Not a concern		118(45.0)	112(45.7)		

*File work: Establish and update health records for rural residents

### Satisfaction

The total satisfaction of both groups was moderate. However, group A reported feeling most satisfied with occupational ecology, followed by life satisfaction, stress, and competency. They reported the least satisfaction with their internal environment (Table 3).

**Table 3:** Job satisfaction of Group A and Group B

Dimension	Group A	Group B	*Z*	*P*-value
Internal environment	2.81 ± 0.687	2.87 ± 0.611	−1.231	0.218
Life satisfaction	3.06 ± 0.699	3.04 ± 0.628	−0.078	0.937
Competency	3.00 ± 0.654	3.05 ± 0.563	−0.804	0.421
Stress	3.06 ± 0.463	3.06 ± 0.426	−0.291	0.771
Occupational ecology	3.33 ± 0.673	3.26 ± 0.567	−1.446	0.148
Total satisfaction	3.05 ± 0.467	3.06 ± 0.368	−0.162	0.872

Group A members who practiced in central and southern Jiangsu reported higher satisfaction with their internal environment. Income played a crucial role in each dimension except stress. Moderate working hours per week (40–50 h) were positively linked with competency, stress, and total satisfaction. On the other hand, excessive working hours per week (approximately 50 h) were positively related to stress and negatively linked with the internal environment. Attending doctors were more satisfied with their life satisfaction, competency, occupational ecology, and total satisfaction. The post of public health physician was positively correlated with high levels of satisfaction, stress, occupational ecology, and total satisfaction (Table 4).

**Table 4:** Multivariate linear regression of factors associated with satisfaction of Group A

	Internal environment			Life satisfaction			Competency			Stress			Occupational ecology			Total		
	U*B*	*B*	*P*	U*B*	*B*	*P*	U*B*	*B*	*P*	U*B*	*B*	*P*	U*B*	*B*	*P*	U*B*	*B*	*P*
Area Northern JS	0			0			0			0			0			0		
Central JS	0.216	0.144	0.019	0.048	0.032	0.618	0.158	0.111	0.080	0.022	0.021	0.744	0.065	0.044	0.495	0.102	0.100	0.101
Southern JS	0.209	0.132	0.050	0.120	0.075	0.282	0.076	0.050	0.466	0.059	0.055	0.441	− 0.011	−0.007	0.923	0.091	0.084	0.207
Income per month (RMB) < 3000	0			0			0			0			0			0		
3000–6000	0.307	0.219	0.002	0.301	0.212	0.004	0.176	0.132	0.068	−0.011	− 0.012	0.872	0.198	0.145	0.051	0.194	0.204	0.004
≥6000	0.765	0.410	< 0.001	0.713	0.375	< 0.001	0.638	0.359	< 0.001	0.052	0.041	0.604	0.374	0.205	0.009	0.508	0.401	< 0.001
Working hours per week < 40	0			0			0			0			0			0		
40–50	0.169	0.121	0.108	0.082	0.058	0.457	0.227	0.171	0.028	0.200	0.213	0.009	0.181	0.133	0.096	0.172	0.181	0.016
≥50	−0.241	−0.169	0.032	−0.130	−0.089	0.271	0.144	0.106	0.189	0.306	0.319	0.000	0.065	0.046	0.577	0.029	0.030	0.702
Professional title resident	0			0			0			0			0			0		
Attending	0.203	0.120	0.054	0.298	0.173	0.008	0.354	0.220	0.001	0.028	0.024	0.714	0.336	0.203	0.002	0.244	0.212	0.001
post clinician	0			0			0			0			0			0		
PHP*	−0.015	−0.006	0.916	0.207	0.082	0.164	0.146	0.062	0.293	0.317	0.190	0.002	0.439	0.182	0.003	0.219	0.130	0.022

*PHP = Public Health Physician

## Discussion

### Overall job satisfaction

The overall job satisfaction of graduates of the “Rural Oriented Medical Students Training Project” was 2.81 ± 0.687 in Jiangsu. This score was slightly lower than physicians in western provinces of China [15] and the Shandong province [16], and was much lower than their counterparts in Australia and the US [5, 17]. The present study exhibited that 34.0% of graduates were considering quitting their job. This was similar to the 34.03% turnover intention rate reported by a survey for rural doctors in central China [18].

Graduates of the “Rural Oriented Medical Students Training Project” reported the highest levels of satisfaction in occupational ecology (3.33 ± 0.673) and the lowest levels in the internal environment (2.81 ± 0.687). This finding was concurrent with that of a Polish study [19], which exhibited a high level of inherent dimension and a low level of personal dimension in similar items.

### Factors associated with satisfaction

Satisfaction is affected by multiple factors. Income can be a major contributor to dissatisfaction. Compared with their counterparts in the United States, Chinese rural doctors have a much lower income with almost similar work hours [20]. In the national range, wages vary widely with the local economic level. The average annual income of rural physicians in Shanghai was 43,400 RMB [21] (3600 RMB per month), whereas 70% of rural physicians earned less than 2000 RMB per month in the Henan Province [22]^.^ The salary of rural doctors in the Jiangxi and Guangxi Provinces was approximately 1500 RMB per month, whereas in the Qinghai Province was approximately 3500 RMB [23]. In the present study, nearly 60% of rural physicians in Jiangsu earned a monthly income of 3000–6000 RMB, which matched middle- and upper-class salaries nationwide.

The living cost in rural areas is lower than that in urban areas; however, the gap gradually decreases. Additionally, young people in China, including people who worked in rural areas, tend to buy houses downtown. Thus, the commute between town and country also increased the living cost. This could be stressful, especially when the doctors got married or had children. The present study exhibited that an income of 3000–6000 RMB per month was positively linked to the internal environment, life satisfaction, and total satisfaction compared with an income of < 3000 RMB, whereas an income of ≥6000 RMB per month played positive roles in most dimensions of satisfaction except stress. Low income was directly linked to the retention rates of rural physicians. “Higher salary for the same work” was the promise of some rural hospitals. This could attract the project graduate physicians when the workplace is remote or the working environment is poor. The graduates in central and southern Jiangsu were more satisfied with the internal environment than their northern counterparts. When we designed this survey, we divided Jiangsu into three areas according to the economic level, which strongly linked to salary, physician well-being, and rural hospital infrastructure. A lack of highly specialized equipment and a lower financing level might pose barriers to providing complex care [19].

Attending doctors were more satisfied with their internal environment, life satisfaction, competency, occupational ecology, and total satisfaction than residents among graduates of the “Rural Oriented Medical Students Training Project”. This finding is concurrent with that of a Polish study [19]. Studies exhibited the link between satisfaction, age, and length of employment [15, 16, 19, 24]. While professional rank was correlated with age and length of employment, age was not an independent factor of satisfaction, it linked to many factors. This could explain why age did not enter the multiple regression model in the present study. Age, personal qualities, and hard work also play a significant role in attaining a high professional rank. A higher proportion of the project graduates attended outpatient and home visits compared with their counterparts. This explains why attending doctors are more satisfied with competency and occupational ecology. They have their group of patients through years of accumulation, and their patients also recognize their professional services. The critical point for policymakers is to improve the attraction of a rural place and increase the retention rate. “Title promotion” can be used as one of the solutions..

The work hours of rural physicians in Jiangsu were roughly the same as their counterparts in Europe and the US [19, 25]. According to the present study, 55.3% of graduates of the “Rural Oriented Medical Students Training Project” serviced no more than 60 patients per week; however, 36.3% worked for more than 50 h per week. A positive correlation between moderate work hours per week (40–50 h) and high satisfaction on competency, stress, and total satisfaction, higher weekly working hours (≥ 50 h) were positively linked with total satisfaction and satisfaction on work stress. This finding contradicts with other studies [15, 19]. One possible explanation for this is that graduates of the “Rural Oriented Medical Students Training Project” comprised a younger population. Thus, they are on a rising period of their career. Working hours accumulate working experience and reflect the value of their job, which also builds professional confidence. Lu et al. [24] mentioned that the retention rate of rural physicians could be affected by recognition of their work.

Public health physicians (PHP) of the graduates of the “Rural Oriented Medical Students Training Project” were more satisfied with occupational ecology and work stress. In China, PHP is mainly responsible for treating infectious diseases and public health emergencies, healthcare of children aged 0–6 years, older adults over 60 years of age, pregnant women, and psychiatric patients [26]. The work content of PHP is relatively fixed. Compared with clinical physicians, PHP exhibited a moderate workload, closer patient-doctor relationships, and a better work reputation.

## Suggestions

Graduates of the “Rural Oriented Medical Students Training Project” exhibited a higher standardized training rate, advanced grade rate, and salary than their peers, meaning that all the promises in the recruitment advertisements have been fulfilled. These policies are crucial in recruiting medical graduates to serve in rural places and should continue to carry forward in the future. But an interesting phenomenon is although the graduates of the “Rural Oriented Medical Students Training Project” showed a “higher salary rate in the same rank” than their peers, their salary is actually no different from that of their peers. The truth is that too few graduates get “higher salary in the same rank”. Satisfaction differences by area and income implied that economic incentives, work hours, professional rank, and post all play significant roles in overall work satisfaction, a series of factors that could affect satisfaction or deeply affect the quality of healthcare services. Appropriate solutions are increasing financial support for graduates of the “Rural Oriented Medical Students Training Project”. This will enhance their professional development opportunities by setting up training centers in rural places and simplifying the promotion mechanism of professional titles.

## Limitation

Because the “Rural Oriented Medical Students Training Project” has just been implemented for 8 years, the graduates were significantly younger than group B physicians, which might lead to differences in marital status and professional rank. Additionally, the cross-sectional nature of the study precludes causal analysis because it is based only on self-reporting, which could encourage socially desirable answers and does not present a causal relationship between the analyzed variables. Furthermore, because this is an investigation linked by private communication and not an executive order, some of the staff might be left out when sending questionnaires. More solutions should be considered to manage this problem in future research. Moreover, due to the sample size, some meaningful factors might not be identified or some meaningless factors wrongly displayed. These biases can be solved through further research with larger sample size.

## Conclusion

Job satisfaction in graduates of the “Rural Oriented Medical Students Training Project” was moderate. Positive factors related to satisfaction include economic incentives, work hours, professional confidence, and post. Increasing financial aid and developing professional identity should be the key points for health administrations to consider.

## Data Availability

The datasets used and analyzed during the current study are available from the corresponding author on reasonable request.

## Abbreviations

GDP: Gross National Product
PCA: Principal Component Analysis
KMO: Kaiser–Meyer–Olkin
SD: Standard Deviation
TCM: Traditional Chinese Medicine
PHP: Public Health Physician
